# Efficacy of an organophosphorus hydrolase enzyme (OpdA) in human serum and minipig models of organophosphorus insecticide poisoning

**DOI:** 10.1080/15563650.2019.1655149

**Published:** 2019-08-27

**Authors:** Michael Eddleston, R. Eddie Clutton, Matthew Taylor, Adrian Thompson, Franz Worek, Harald John, Horst Thiermann, Colin Scott

**Affiliations:** a Department of Pharmacology, Toxicology & Therapeutics, University/BHF Centre for Cardiovascular Science, University of Edinburgh, Edinburgh, UK;; b Department of Anaesthesia, Royal (Dick) School of Veterinary Studies, University of Edinburgh, UK;; c CSIRO Biocatalysis & Synthetic Biology Team, Black Mountain Science and Innovation Park, Canberra, Australia;; d Bundeswehr Institute of Pharmacology and Toxicology, Munich, Germany

**Keywords:** Organophosphorus insecticides, antidotes, treatment, enzymes, animal model

## Abstract

**Objectives:** Current therapeutic options for organophosphorus (OP) insecticide self-poisoning including atropine and oximes are inadequate and case fatality may exceed 20%. An OP hydrolase enzyme, OpdA, has been used for environmental cleansing of OP insecticides and prevented death in rat and non-human primate models of OP insecticide poisoning if given very quickly after exposure. We here tested OpdA’s ability to break down OP insecticides in human serum and in clinically relevant minipig models of OP insecticide poisoning.

**Methods:** Human serum was spiked with seven diverse WHO Class II OP insecticides (chlorpyrifos, quinalphos, diazinon, dimethoate, fenthion, phenthoate, and profenofos) and the effect of OpdA on degradation measured. The pharmacodynamic and clinical effects of OpdA treatment were studied in Gottingen minipigs orally poisoned with agricultural formulations of dimethoate EC40 or methyl parathion EC60; pharmacodynamic effects were also assessed in profenofos EC50-poisoned pigs.

**Results:** OpdA effectively hydrolysed OP insecticides in human serum, with rates varying from 856 (SD 44) down to 0.107 (SD 0.01) moles of substrate hydrolysed/mole of enzyme/sec (k_cat_) for quinalphos and phenthoate, respectively, although at rates 2–3 log orders less than found *in vitro* in buffered solution. It showed clinical benefit in minipig models, reducing the dose of noradrenaline required to sustain an adequate mean arterial pressure after dimethoate (mean 0.149 [SD 0.10] μg/kg/h vs. 1.07 [SD 0.77] μg/kg/h, *p* < .0001) and methyl parathion (mean 0.077 [SD 0.08] μg/kg/h vs. 0.707 [SD 0.49] μg/kg/h, *p* < .0001) poisoning. OpdA reduced blood OP insecticide concentration and acetylcholinesterase inhibition after poisoning by dimethoate, methyl parathion, and profenofos insecticides.

**Conclusions:** *In vitro* incubation of OpdA in human serum showed hydrolysis of diverse OP insecticides, although at lower rates than found in buffer solutions. This activity results in clinical and pharmacodynamic efficacy *in vivo* against several OP insecticides. These results support the testing of OpdA in further animal models before considering human trials to determine whether it may become an urgently required novel therapeutic agent for OP insecticide self-poisoning.

## Introduction

Organophosphorus (OP) insecticide poisoning kills over 200,000 people each year, mostly following self-harm, in rural Asia [1,2]. These compounds inhibit multiple enzymes, in particular, acetylcholinesterase (AChE) and butyrylcholinesterase (BuChE) [3–5]. While BuChE inhibition appears unimportant in acute poisoning, AChE inhibition results in overstimulation of acetylcholine receptors in the autonomic nervous system, neuromuscular junction (NMJ), and central nervous system [3]. This syndrome is termed the cholinergic crisis and causes acute respiratory and cardiovascular failure. Self-poisoning is also complicated by the presence of large amounts of solvents which are themselves toxic [6]. Many deaths occur within hours of pesticide ingestion during the acute cholinergic crisis, usually due to acute respiratory failure that occurs before mechanical lung ventilation is instigated [7]. Other deaths occur later, in ventilated patients due to cardiovascular collapse [8], failure of NMJ transmission, or complications of aspiration [9,10].

Standard treatment of acute OP poisoning involves resuscitation, and the administration of oxygen, the muscarinic antagonist atropine, and an oxime AChE reactivator such as pralidoxime or obidoxime [4,11,12]. Unfortunately, this standard therapy is frequently ineffective, with many patients who survive to hospital presentation dying after admission [10,13]. Respiratory failure does not respond to atropine, often requiring tracheal intubation and mechanical lung ventilation for several weeks [9]. Clinical trials of pralidoxime do not show consistent clinical benefit [14–16]. Overall, medical management is difficult in the Asian district hospitals that admit the majority of patients [17]. There are too few intensive care resources and effective treatments as well as too little good evidence with which to improve treatment for patients after ingestion of these highly toxic compounds [18].

The aryldialkylphosphatase (EC 3.1.8.1), or OP hydrolase enzyme, OpdA has been isolated from the bacterium *Agrobacterium radiobacter* P230. It is highly effective at hydrolysing OP pesticides and G-series nerve agents (e.g., soman) *in vitro* [19,20] and in contaminated agricultural water (OpdA fixed to a matrix structure cleared > 90% of methyl parathion from 84,000 L of fast-flowing water in just 10 min in a field trial [21]). Compared with a range of other OP hydrolases [22,23], it has excellent *in vitro* activity against a wide range of pesticides that might be encountered in human poisoning.

The addition of OpdA to usual clinical treatment might improve the effectiveness of treatment for OP pesticide poisoning, in particular allowing oximes to work in mega-dose suicides [24]. It prevents death in rats when given immediately after dosing with highly toxic dichlorvos, parathion or methyl parathion OP insecticides, improving the effectiveness of pralidoxime [25], and prevents acute toxicity after oral dichlorvos poisoning in non-human primates [26,27]. However, before development can start, additional studies are required. In these studies, we aimed to assess its function in human serum since it is uncertain how it will interact with components of serum and to test its efficacy at hydrolysing OP insecticides in more relevant *in vivo* models to allow broader assessment than possible from rodent studies. Evidence of hydrolysis in these studies would support the production of an enzyme to Good Manufacturing Practice standards that could be used for further studies and progression into pre-clinical toxicology and human studies.

## Methods

OpdA was produced by CSIRO at a concentration of 8.1 mg/mL in saline. Shake flask cultures produce ∼50 mg of pure enzyme per litre of the medium [28]. OpdA was kept under conditions under which it had previously been shown to be stable; the enzyme was pure by Coomasie-stained SDS/PAGE.

### Serum studies

The following OP insecticides were selected to represent a range of WHO Class II toxicity OP insecticides [29] from three different OP chemical classes: diethyl (chlorpyrifos, quinalphos, diazinon), dimethyl (dimethoate, fenthion, phenthoate), and S-alkyl (profenofos) [30]. We selected Class II toxicity OP insecticides since the Food and Agriculture Organisation (FAO) has recommended withdrawing all WHO Class I toxicity pesticides from agricultural practice [31,32]; WHO Class II OP insecticides are, therefore, becoming more important in agriculture and in poisoning over time.

OpdA activity assays against OP insecticide substrates were conducted in pooled human serum spiked with 500 µM substrate and 100 µM internal standard (3-chloroaniline and thiobencarb) at 25 °C. A control with no enzyme was run with each experiment and the background-subtracted to account for non-specific pesticide degradation. Data on activity in the buffer (50 mM Tris-HCl, pH 8.0) was taken from previously collected unpublished CSIRO data.

Samples were taken at time points from 0 to 16 min with a minimum of 30 sec interval between samples. At each time point, 100 µL of sample was removed from the 5 mL incubation mixture, stopped with an equal volume of toluene, the OP insecticides extracted into the organic phase by vortexing for 2 min followed by centrifugation at 14,000 *g* at 4 °C, and then analysed by gas chromatography-mass spectrometry (GC-MS) on an Agilent GC-MS as per previously published methods [33]. Reference OP insecticide compounds were obtained from Sigma-Aldrich as Pestanal standards (>99% pure). Enzyme concentrations varied from 2 nM to 2 µM, and samples were collected at intervals to enable at least seven time points with a linear loss of substrate. All assays were repeated in triplicate. The rate of reaction was calculated by loss of substrate using SigmaPlot (Systat software, Germany).

### Pig studies

After institutional ethical review, studies were performed under UK Home Office Licence in 30 adult male Göttingen minipigs (Ellegaard Minipigs ApS, Dalmose, Denmark) with a mean weight of 19.7 (SD 3.6) kg. Animals were drug-naïve and barrier bred and shown to be free of infections before shipment. Animals were treated in accordance with the Animals (Scientific Procedures) Act of 1986

### Study design

The individual animal was the experimental unit. Bias was minimised by randomly allocating animals to treatment using a random number list. The allocation could not be predicted; the study was an open study but the outcomes were robust and not likely to be affected by bias [34].

### Anaesthesia

Animals were kept in pens with free access to food scattered in their bedding and water under the care of institutional veterinary surgeons. Food was withheld for one night before each study. Pre-anaesthetic medication was intramuscular ketamine (5 mg/kg) and midazolam (0.5 mg/kg). Anaesthesia was induced with 5% isoflurane (selected since the effect of this anaesthetic on AChE activity is well-characterized [35]) in oxygen delivered via facemask. The trachea was intubated and anaesthesia maintained to a clinically acceptable depth using isoflurane in oxygen delivered initially via a circle breathing system. Intermittent positive pressure ventilation (IPPV) was provided as necessary using a minute volume divider (Manley Pulmovent, Harlow, UK) adjusted to maintain normocapnia.

Inspired and expired carbon dioxide, oxygen and isoflurane concentrations were monitored. Heart rate, oesophageal and peripheral temperature, electrocardiogram, and percentage of saturated haemoglobin were recorded (Datex, Clearwater, FL). The temperature was maintained as close to physiological values as possible by the use of forced warm air blankets (Bair Hugger, Arizant, Wakefield, UK), heat pads and high ambient temperature. Ten millilitre per kilogram per hour Ringer’s lactate solution was administered for the first 30 min after induction of anaesthesia and then at 5 ml/kg/h for the remainder of the study. Fluid administration was increased as necessary to maintain urine output and optimize central venous pressure.

### Instrumentation and monitoring

A central arterial catheter was placed surgically into the carotid artery for continuous arterial pressure monitoring and blood sampling. A central venous catheter was placed into the external jugular vein for infusion of drugs and monitoring of central venous pressure. The catheters were connected to a pressure manometer (Datex, Clearwater, FL) zeroed at the level of the heart base to give arterial and CVP pressure data. An orogastric tube was placed for poison gavage and the bladder catheterised by surgical cutdown.

### OP insecticides

Pilot minipig studies of poisoning with commercial formulations of WHO toxicity class II dimethoate and chlorpyrifos insecticides showed that the latter did not cause clinical effects or inhibition of AChE (unpublished observations), despite the use of doses equivalent to those believed to cause substantial human poisoning [8]. We, therefore, selected a potent WHO Class I toxicity fat-soluble insecticide, methyl parathion, for clinical studies alongside dimethoate, and assessed OpdA’s pharmacodynamic effect on profenofos poisoning. Commercial formulations (including solvents and other adjuvants) used for these studies were dimethoate EC40 (emulsifiable concentrate [36] at 400 g/L of active ingredient [AI]) (dose: 2.5 mL/kg, 1000 mg/kg of AI; BASF SE, Ludwigshafen, Germany), methyl-parathion EC60 (dose: 2 mL/kg, 1200 mg/kg AI; Cheminova, Lemvig, Denmark), and profenofos EC50 (dose: 3 mL/kg, 1500 mg/kg AI; Syngenta, Basel, Switzerland). These AI doses markedly exceed the rat oral LD50s of 150 mg/kg, 14 mg/kg, and 358 mg/kg, respectively, for each insecticide [29].

### Experimental protocol

After arterial catheter insertion, 60 min passed before poisoning during which time baseline observations were recorded. Minipigs were then administered insecticide by oral gavage followed by 60 mL of water at room temperature.

In the initial control and dimethoate groups, OpdA was administered intravenously (IV) as a single 0.8 mg/kg dose over 1 h, starting 1 h post poisoning. After noting that the effect of OpdA appeared to be transient, it was subsequently administered as a 0.4 mg/kg IV loading dose over 1 h, starting 1 h post poisoning, followed by 0.4 mg/kg as an infusion over 8 h for the methyl parathion and profenofos groups in an attempt to improve its efficacy. Atropine (30 mcg/kg) was administered IV as required for muscarinic effects (increased secretions); pralidoxime was not administered in this model. Noradrenaline was administered IV to maintain a mean arterial pressure (MAP) greater than 55 mmHg, with a target MAP of 65 mmHg.

The study was ended by euthanasia using IV pentobarbital or anaesthetic overdose after 12 h, or when the MAP fell below 45 mmHg and could not be restored with fluids or vasopressor.

### Measurements

Cardiovascular data were collected 30 and 10 min before poisoning and 15 min intervals thereafter using a Datex monitor. Arterial blood samples were taken at −40, −10, and 30 min, and then every hour, and lactate analysed using an i-STAT (Abbott, Princeton, NJ). Analyses for plasma BuChE and red cell AChE activity were performed as previously described [8,37]. Organophosphorus insecticides were detected by LC-ESI-MS/MS and FI-ESIMS/MS-based on the methods of Salm and colleagues [38] using a PE 200 series for chromatography (Perkin Elmer, Rodgau-Jügesheim, Germany) online coupled to 4000 QTrap triple quadrupole MS (AB Sciex, Darmstadt, Germany) [39].

### Statistical analysis

Primary data analysis was conducted in Prism 7.0 (GraphPad, San Diego, CA). All animals were included in the analysis. Pig weights and clinical/biochemical outcomes were summarised with mean and SD or SE; means were compared using the *t*-test.

## Results

### In vitro studies of OpdA in human serum

**Figure 1.**
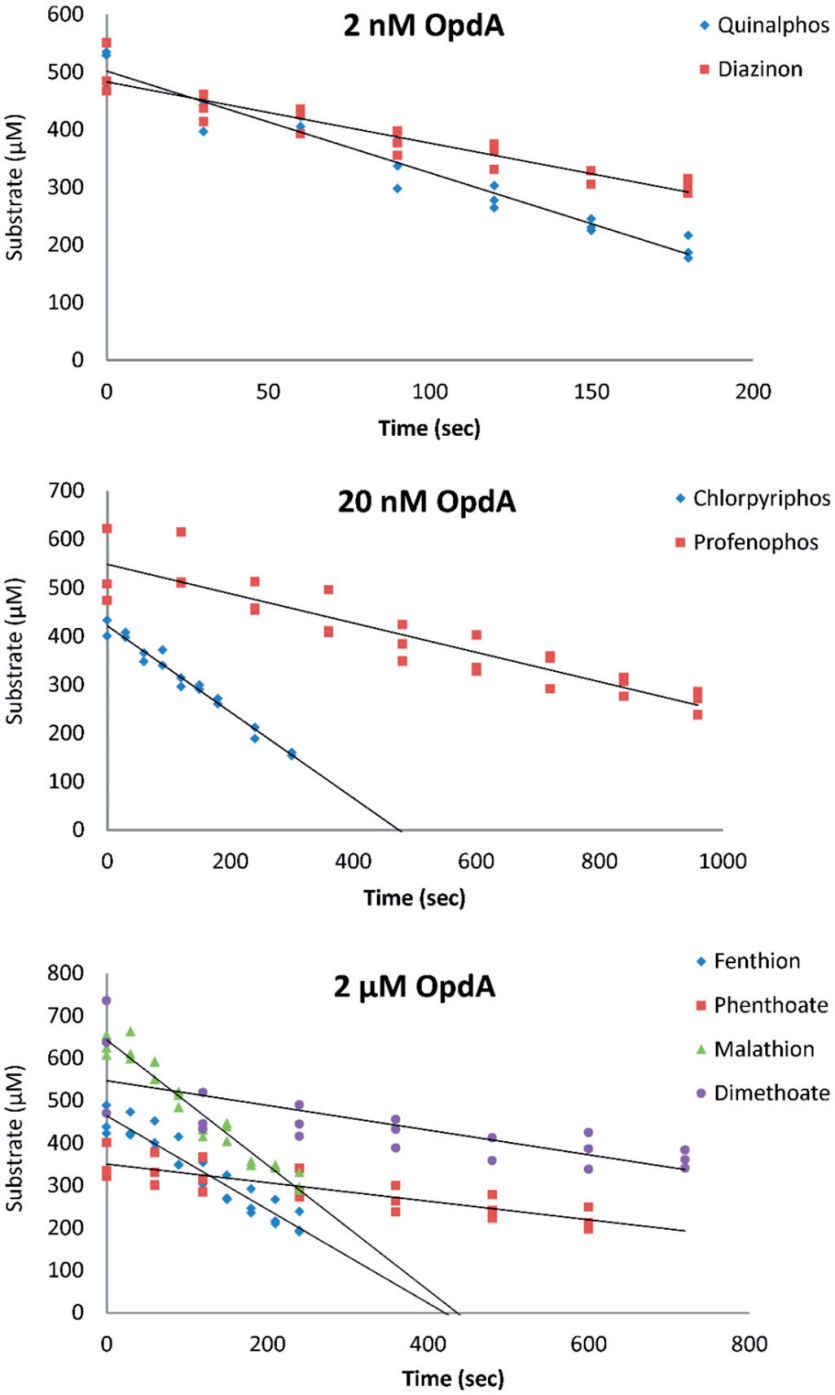
*In vitro* determination of enzyme kinetics in human serum. Determination of the rate of OP insecticide hydrolysis by 2 nM to 2 μM OpdA for eight OP insecticides.

We first assessed the ability of OpdA to hydrolyse diverse WHO toxicity class II OP insecticides in human serum. OpdA was able to hydrolyse all the insecticides, although at markedly different rates and at lower rates than in buffer (Figure 1, Table 1).

**Table 1.** Activity of OpdA enzyme in human serum and in buffer against WHO Class II OP insecticides.

Pesticide	Chemistry	Activity in human serum (mol/sec/ mol enzyme)	Activity in buffer (mol/sec/ mol enzyme)
Quinalphos	Diethyl	856 ± 44	190,000
Diazinon	Diethyl	465 ± 34	150,000
Chlorpyrifos	Diethyl	58.3 ± 1.5	18,000
Profenofos	S-alkyl	15.3 ± 1.3	ND
Malathion	Dimethyl	0.833 ± 0.049	48
Fenthion	Dimethyl	0.406 ± 0.027	ND
Dimethoate	Dimethyl	0.161 ± 0.025	9.0
Phenthoate	Dimethyl	0.107 ± 0.010	ND

The buffer activity data are unpublished from CSIRO Entomology; the buffer was 50 mM Tris-HCl (pH 8.0). ND: not done. Human serum data are mean ± SD, *n* = 3.

The diethyl OP quinalphos was most rapidly hydrolysed with a rate of 856 moles of substrate hydrolysed/mole of enzyme/sec (k_cat_); the dimethyl OPs phenthoate and dimethoate were least well hydrolysed with rates of 0.1 and 0.2 k_cat_, respectively. Overall, satisfactory activity was shown for the three diethyl OP pesticides. Profenofos, an S-alkyl OP, was hydrolysed with intermediate efficiency while the four dimethyl OP pesticides were least efficiently hydrolysed (Table 1).

### In vivo studies of OpdA in dimethoate EC poisoned minipigs

We have established a Gottingen minipig model of oral poisoning with 2.5 mL/kg of the 40% agricultural dimethoate emulsifiable concentrate formulation (EC40) which causes early respiratory failure, distributive shock, lethal cardiovascular collapse, and severe AChE inhibition [6]. These features are similar to human self-poisoning with comparable doses of dimethoate EC40 [8,40].

**Figure 2.**
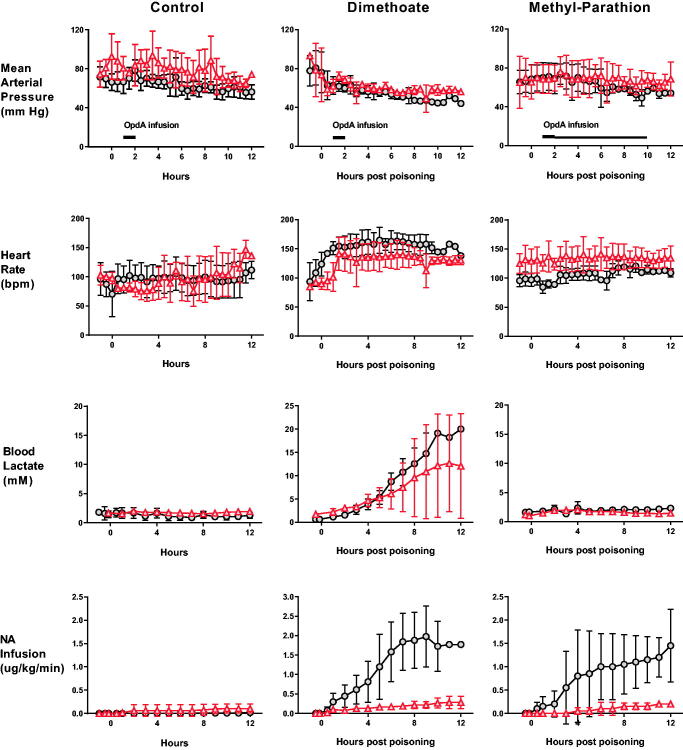
Clinical effect of OpdA on OP insecticide poisoning. Heart rate, mean arterial pressure, arterial blood lactate concentrations, and noradrenaline requirements in pigs after poisoning with dimethoate EC40 (*n* = 9), methyl-parathion EC60 (*n* = 4), or saline placebo (*n* = 9), with (triangles) or without (circles) OpdA. Note that the dosing of OpdA varied between the control and dimethoate EC40 studies and the methyl parathion EC60 study, in which the duration of therapy infusion was extended (from total dose over 1 h, to half dose of 1 h and half dose over 8 h). OpdA dosing was started 1 h after oral gavage. The timing of administration is indicated by black bars in the mean arterial pressure panels. Values are group means ± SD.

We first tested OpdA in this model of dimethoate poisoning. Animals received 2.5 mL/kg dimethoate EC40 by oral gavage followed by (or not) OpdA at 1 h post-poisoning (0.8 mg/kg infused IV over 1 h). Pigs that received OpdA required less noradrenaline to maintain their MAP over the 12 h (mean 0.149 [SD 0.10] μg/kg/h vs. 1.07 [SD 0.77] μg/kg/h, *p* < .0001; Figure 2).

**Figure 3.**
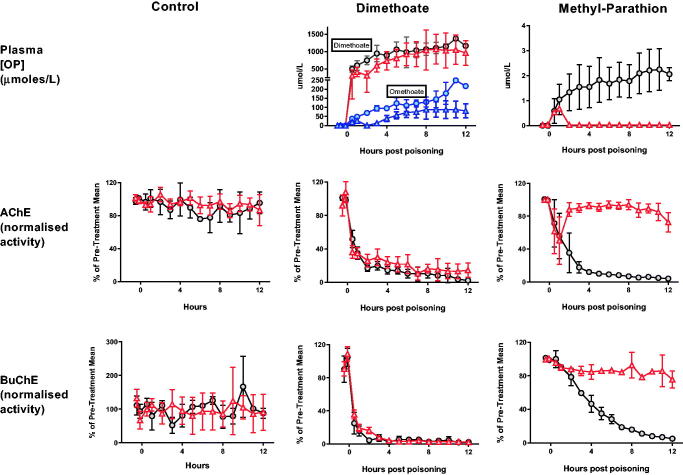
Toxicodynamic effect of OpdA on OP insecticide poisoning. Plasma OP insecticide concentration, red cell AChE activity, and plasma BuChE activity in pigs after poisoning with dimethoate EC40, methyl-parathion EC60, or saline placebo, with or without OpdA. Symbols as in Figure 2 with addition of omethoate concentrations with (triangles) or without (circles) OpdA. Note that the dosing of OpdA varied between the control and dimethoate EC40 studies and the methyl parathion study, increasing the duration of therapy infusion in the latter (from total dose over 1 h, to half dose of 1 h and half dose over 8 h). OpdA dosing was started 1 h after oral gavage. Values are group means ± SD.

Despite relatively poor hydrolysis of dimethyl OPs (Table 1), OpdA hydrolysed both dimethoate and its active metabolite omethoate, causing a reduction in their concentration after 1 h (Figure 3). The effect was sustained for omethoate out to 12 h (mean AUC without OpdA 1480 [SE 68.8] μmol/L*h vs. 682.1 [78.4] μmol/L*h with OpdA; *p* < .001). In contrast, the effect on the higher concentrations of dimethoate was not sustained (mean AUC without OpdA 11,691 [SE 471] μmol/L*h vs. 9495 [852] μmol/L*h with OpdA; *p* = .0649). There was a small reduction in AChE inhibition after OpdA treatment but minimal effect on BuChE inhibition at 2 h (Figure 3).

### In vivo studies of OpdA in a minipig model of methyl parathion EC poisoning

We then tested OpdA against the highly toxic fat-soluble dimethyl OP, methyl parathion. To increase the possibility of sustained OpdA effect, we used a revised IV dosing regimen that involved giving half the dose in the first hour, starting 1 h post poisoning, and the second half over the subsequent 8 h (0.4 mg/kg over 1 h, followed by 0.4 mg/kg over the following 8 h).

Unlike dimethoate poisoning, methyl parathion poisoning in the pig under terminal anaesthesia did not cause major clinical effects (Figure 2) which was surprising in light of human cases in which substantial poisoning develops within minutes to hours [41,42]. Inhibition of both AChE and BuChE occurred slowly. OpdA treatment resulted in a reduced noradrenaline requirement to sustain predetermined blood pressure values (mean 0.077 [SD 0.08] μg/kg/h vs. 0.707 [SD 0.49] μg/kg/h, *p* < .0001; Figure 2).

OpdA was highly active against methyl parathion, causing rapid and complete hydrolysis of the compound in the circulation (mean AUC without OpdA 20.3 [SE 1.8] μmol/L*h vs. 0.79 [0.06] μmol/L*h with OpdA; *p* < .01). Reactivation of both red cell AChE (mean AUC over 12 h of AChE normalised against baseline: 1080 [SE 25.2] vs. 267 [SE 26.6], *p* < .01) and plasma BuChE (mean AUC over 12 h of BuChE normalised against baseline: 1084 [SE 19.7] vs. 503 [SE 13], *p* < .01) occurred, which was sustained until the end of the infusion (Figure 3).

### Pharmacodynamic effect of OpdA in a minipig model of profenofos EC poisoning

We also tested the effect of OpdA in a minipig model of oral profenofos EC40 self-poisoning. Unexpectedly, despite clear inhibition of both AChE and BuChE, profenofos poisoning did not cause apparent clinical toxicity in the minipigs.

**Figure 4.**
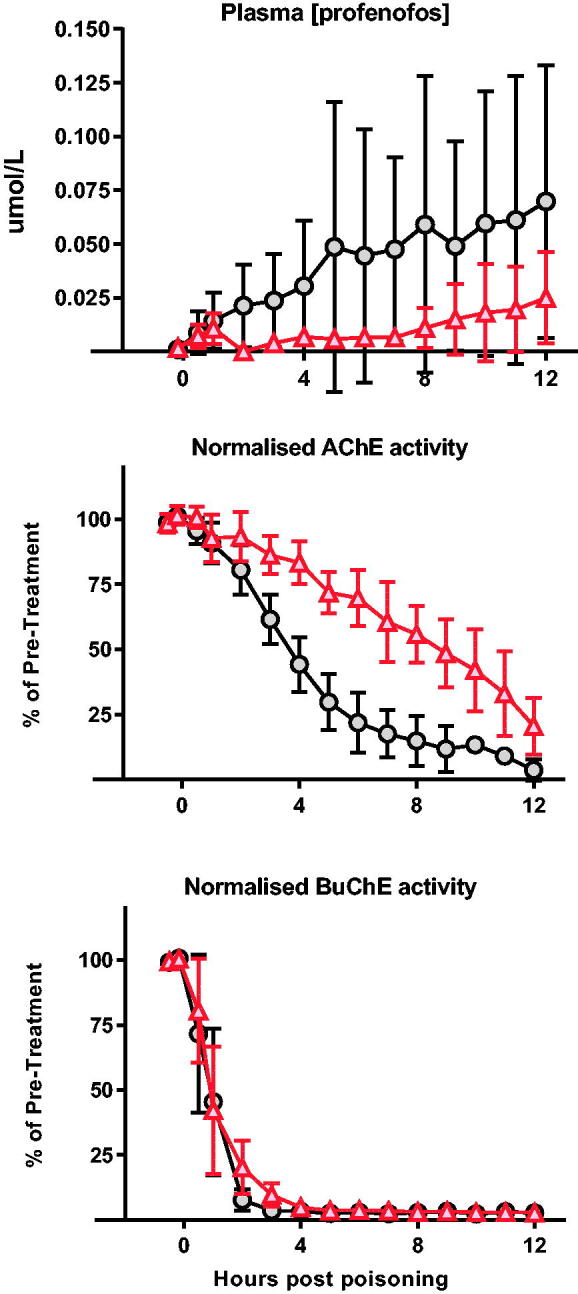
Pharmacodynamic effect of OpdA on profenofos poisoning. Plasma profenofos concentration, red cell AChE activity, and plasma BuChE activity in pigs (*n* = 8) after poisoning with profenofos EC50, with or without OpdA. OpdA was started 1 h after oral gavage and administered as half dose of 1 h and half dose over 8 h. Due to the lack of clinical effect of profenofos in anaesthetised pigs, noradrenaline was not required as a vasopressor. Symbols as for Figure 2. Values are group means ± SD.

We, therefore, assessed the pharmacodynamic effect of OpdA when given one hour after oral gavage of profenofos (Figure 4). An IV loading dose of OpdA followed by an 8 h infusion resulted in a substantially lower blood profenofos concentration over the 12 h (mean AUC without OpdA 0.496 [SE 0.12] μmol/L*h vs. 0.119 [0.03] μmol/L*h with OpdA; *p* = .023). This was associated with slower AChE inhibition (mean AUC over 12 h of AChE normalised against baseline: 850 [SE 27.9] vs. 497 [SE 20.7], *p* < .0001) but a minimal difference in BuChE activity (mean AUC over 12 h of BuChE normalised against baseline: 204 [SE 18.1] vs. 176 [SE 20.7], *p* = .373) (Figure 4).

## Discussion

This study has shown that OpdA can hydrolyse a variety of OP pesticides in human serum and that its IV administration at a clinically relevant time point in a large animal model is able to reduce plasma insecticide concentrations, retard AChE inhibition, and for two OP insecticides (dimethoate and methyl parathion) offer clinical benefit as shown by reduced requirements for vasopressor support.

These results indicate that OpdA may have a clinical role in treating patients with OP insecticide poisoning and may improve the efficacy of other therapies, such as oximes. Treatment as early as 1 h post-ingestion is feasible since a substantial proportion of patients in Asia (where most patients occur and where this antidote is most required) present to primary rural hospitals within this time frame. If the antidote can be made affordable to Asian health care systems, and its use shown to be associated with few significant adverse reactions, then it could be used immediately as a matter of routine in all patients presenting to primary rural hospitals. OpdA administration before the onset of clinical signs would offer the best opportunity of breaking down the OPs before they cause severe illness.

Technically, it may become feasible to rapidly identify the OP insecticide to which a patient has been exposed (determining whether the OP is a good substrate for OpdA) before deciding to give the antidote. However, such approaches are probably many years away from routine clinical use, and even modest hydrolysis may be sufficient to offer clinical benefit to patients.

The highly efficient production of OpdA in bacteria suggests that it will be possible to produce it affordably (akin to streptokinase) although its immunogenicity and subsequent risk of adverse reactions would limit its subsequent use in patients over the following 12 months (again akin to streptokinase) [43]. Human safety, tolerance, and immunogenicity testing will be required prior to clinical efficacy studies.

Activities of tens to hundreds of OP molecules hydrolysed per second are excellent *in vivo* activities for enzymes in human serum (for example, streptokinase has a k_cat_ of 22), although there was variation between insecticides as expected due to steric complementarity between the substrate and substrate-binding pocket [44]. Despite the activity in human serum being good, it was markedly (2–3 logs) less active than found in buffer.

A number of factors are well known to influence enzyme activity including pH, temperature, and the ionic strength of the buffer [45]. Human serum is a complex mixture of proteins, lipids, metabolites, and salts and all of these elements may interact with both the enzyme and substrate. Such interactions may inhibit enzyme activity through competitive binding at active site or changing protein structure. Similarly, serum components such as albumin may interact with the substrate and decrease the available concentration, reducing enzyme activity [46].

However, this activity was sufficient for effect, showing reduced plasma OP insecticide concentrations in all treated animals, but especially against methyl parathion which showed remarkably rapid hydrolysis and consequently reactivation of inhibited AChE due to reduced inhibitor and spontaneous reactivation. The high activity was expected from previous findings of a relatively high k_cat_ *in vitro* [19] and highly effective hydrolysis of methyl parathion in agricultural run-off water [21]. Of note, the rates of inhibition of both AChE and BuChE by methyl parathion were slow, as has been noted in a human poisoning case [47], possibly due to slow *in vivo* conversion from methyl parathion to methyl paraoxon.

Treatment of dimethoate poisoned pigs showed clinical benefit (reduced requirements for noradrenaline) associated with lower plasma concentrations of the active metabolite omethoate. However, this was associated with no reduced inhibition of BuChE and very little change in inhibition of AChE. This raises again questions about the utility of using red cell AChE and plasma BuChE to assess the severity of acute OP insecticide poisoning [48].

### Limitations

The study was designed to assess proof of principle and did not test OpdA against hard endpoints such as survival. This would have required much larger studies than planned here but can be considered for the next stage. The dosing of OpdA varied between the control/dimethoate studies and methyl parathion/profenofos studies, increasing the duration of effective therapy in the latter. As a result, the dimethoate and methyl parathion/profenofos studies cannot be directly compared, although both show pharmacokinetic and pharmacodynamic effect.

The clinical benefit of OpdA remains unclear: although the enzyme reduced vasopressor requirements after dimethoate poisoning in minipigs, there was no major effect on arterial lactate concentration, a marker of tissue hypoxia and overall toxicity. Furthermore, poisoning with methyl parathion and profenofos was markedly less severe in pigs than humans, with the pigs remaining well despite marked AChE inhibition and doses well in excess of the rat oral LD50. These findings limit the usefulness of minipig models for determining the likely efficacy of OpdA in humans. A further limitation is the use of isoflurane anaesthesia in the pigs during the study since this is quite different from the situation with poisoned humans. However, we chose isoflurane as this agent has a well-described, consistent and modest effect on cholinesterase activity [35]. There is also no indication that isoflurane will have affected OpdA action. The toxicokinetics should be an objective and robust readout of effect.

It will be important to assess whether OpdA hydrolyses the active oxon metabolites of thion OP insecticides such as parathion. However, in this study, we did not measure the breakdown of the oxons either *in vitro* or *in vivo*. Further studies are required to assess this point.

We did not perform *in vitro* studies of methyl parathion in human plasma and so could not compare activity *in vivo* in the pig with *in vitro* activity. We also did not perform *in vitro* studies with minipig plasma that might have been informative about the expected *in vivo* human activity. These can be considered for future studies if the enzyme moves into clinical development; their absence should not hinder this decision making.

In conclusion, we have shown that OpdA breaks down OP insecticides in human serum and in poisoned pigs and that this offers clinical and pharmacodynamic benefits. The rat, pig and primate studies now support the idea of progressing OpdA through a program of safety and efficacy trials with the objective of gaining regulatory approval for its use as an antidote. It is possible that it may become an effective addition to the pharmacological armamentarium for this important cause of poisoning and suicide worldwide.
